# Reicher

**Published:** 1912-02

**Authors:** 


### ABSTRACTS

REICHER, quoted from *Therapeutic Gazette,* October 15, 1911, recommends 1—1½ c.c. of 1:1000 adrenalin solution containing ½ per cent. novacaine, injected every two or three days into cancer. He claims good results from the shriveling of the mass. Naturally, at present such treatment should be limited to inoperable cases and may we suggest watching the effects on blood pressure, especially in the elderly among whom most cancers occur. The joint use of nitrites should counteract any danger of excessive blood pressure. As to remote effects on blood vessels, the pancreas, etc., the local indication justifies almost any risk.

